# Chemical rules for optimization of chemical mutagenicity via matched molecular pairs analysis and machine learning methods

**DOI:** 10.1186/s13321-023-00707-x

**Published:** 2023-03-20

**Authors:** Chaofeng Lou, Hongbin Yang, Hua Deng, Mengting Huang, Weihua Li, Guixia Liu, Philip W. Lee, Yun Tang

**Affiliations:** Shanghai Frontiers Science Center of Optogenetic Techniques for Cell Metabolism, School of Pharmacy, East China University of Science and Technology, Shanghai, 200237 China

**Keywords:** Mutagenicity optimization, Lead optimization, Matched molecular pairs analysis, Machine learning, Consensus model

## Abstract

Chemical mutagenicity is a serious issue that needs to be addressed in early drug discovery. Over a long period of time, medicinal chemists have manually summarized a series of empirical rules for the optimization of chemical mutagenicity. However, given the rising amount of data, it is getting more difficult for medicinal chemists to identify more comprehensive chemical rules behind the biochemical data. Herein, we integrated a large Ames mutagenicity data set with 8576 compounds to derive mutagenicity transformation rules for reversing Ames mutagenicity via matched molecular pairs analysis. A well-trained consensus model with a reasonable applicability domain was constructed, which showed favorable performance in the external validation set with an accuracy of 0.815. The model was used to assess the generalizability and validity of these mutagenicity transformation rules. The results demonstrated that these rules were of great value and could provide inspiration for the structural modifications of compounds with potential mutagenic effects. We also found that the local chemical environment of the attachment points of rules was critical for successful transformation. To facilitate the use of these mutagenicity transformation rules, we integrated them into ADMETopt2 (http://lmmd.ecust.edu.cn/admetsar2/admetopt2/), a free web server for optimization of chemical ADMET properties. The above-mentioned approach would be extended to the optimization of other toxicity endpoints.

**Graphical Abstract:**

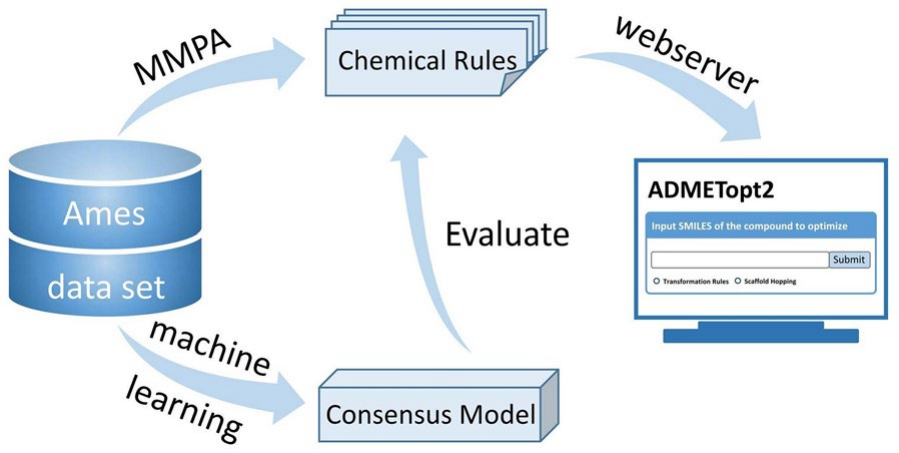

**Supplementary Information:**

The online version contains supplementary material available at 10.1186/s13321-023-00707-x.

## Introduction

Chemical mutagenicity is a serious issue to be addressed in early drug discovery [1, 2]. More specifically, gene mutation caused by a compound is a permanent and irreversible change closely related to carcinogenicity, which is a great threat to human health [3]. The most popular in vitro test system to assess potential mutagenic potency of a compound is the bacterial reverse mutation test called the Ames assay [4, 5]. It uses the mechanism of back mutation in different bacteria strains, typically *Salmonella typhimurium*, to detect different types of mutations. The early-stage detection of chemical mutagenicity is of great significance for increasing the effectiveness of drug development [6]. However, with the rapid expansion of the chemical space explored by medicinal chemists, large-scale in vitro assays are not feasible considering labor, time and cost. In addition, the rising amount of data makes it more difficult to manually extract chemical rules related to the optimization of mutagenicity. Therefore, researchers proposed many computational algorithms to automatically learn hidden chemical knowledge from large data sets and developed many valuable computational tools [7–9]. These technologies offer cheaper and faster alternatives for the evaluation and optimization of chemical mutagenicity and have been recognized by many international organizations [10].

In the past decade, many machine learning models for mutagenicity prediction had been proposed and obtained favorable predictive performance [6, 11, 12]. In general, these models could be roughly divided into two categories: conventional machine learning models and deep learning models. The former utilized molecular descriptors, such as molecular fingerprints and physicochemical properties, combined with conventional machine learning algorithms, such as random forest (RF) [13] and support vector machine (SVM) [14], to build models [7, 15]. The latter preferred to use molecular graphs and multilevel network architectures for model construction [9, 16]. Conventional machine learning models were much simpler but limited by manually selected molecular descriptors and algorithms, whereas deep learning models were more suitable for endpoints with a large amount of data to reduce the risk of falling into the trap of overfitting. However, even though the great achievement of machine learning models in mutagenicity prediction, they could not provide reference guidance for structural modifications of compounds with potential mutagenic effects.

In lead optimization, matched molecular pairs analysis (MMPA) is a powerful tool and is widely used by medicinal chemists to optimize pharmacokinetic properties, toxicity, and physicochemical properties [17–19]. Matched molecular pairs (MMPs) refer to a pair of similar molecules with a single structural change [20]. MMPA aims to derive the chemical rules (i.e., MMP rules) between structural transformations and property changes from MMPs. For example, Paul et al. used MMPA to identify the effect of common substituents on ADMET parameters [19]. Leach et al. utilized MMPA to analyze the effect on aqueous solubility, plasma protein binding, and oral exposure of adding substituents to aromatic rings and methylating heteroatoms [21]. Generally, these valuable chemical rules provide clear design guidance for drug candidates, which reduces the design cycle of drug discovery projects. However, given the complexity of chemical and biological systems, the same substitution of different molecules might result in different property changes [17, 22]. Therefore, it is necessary to evaluate the generalizability and validity of MMP rules when applied to different molecules.

**Fig. 1:**
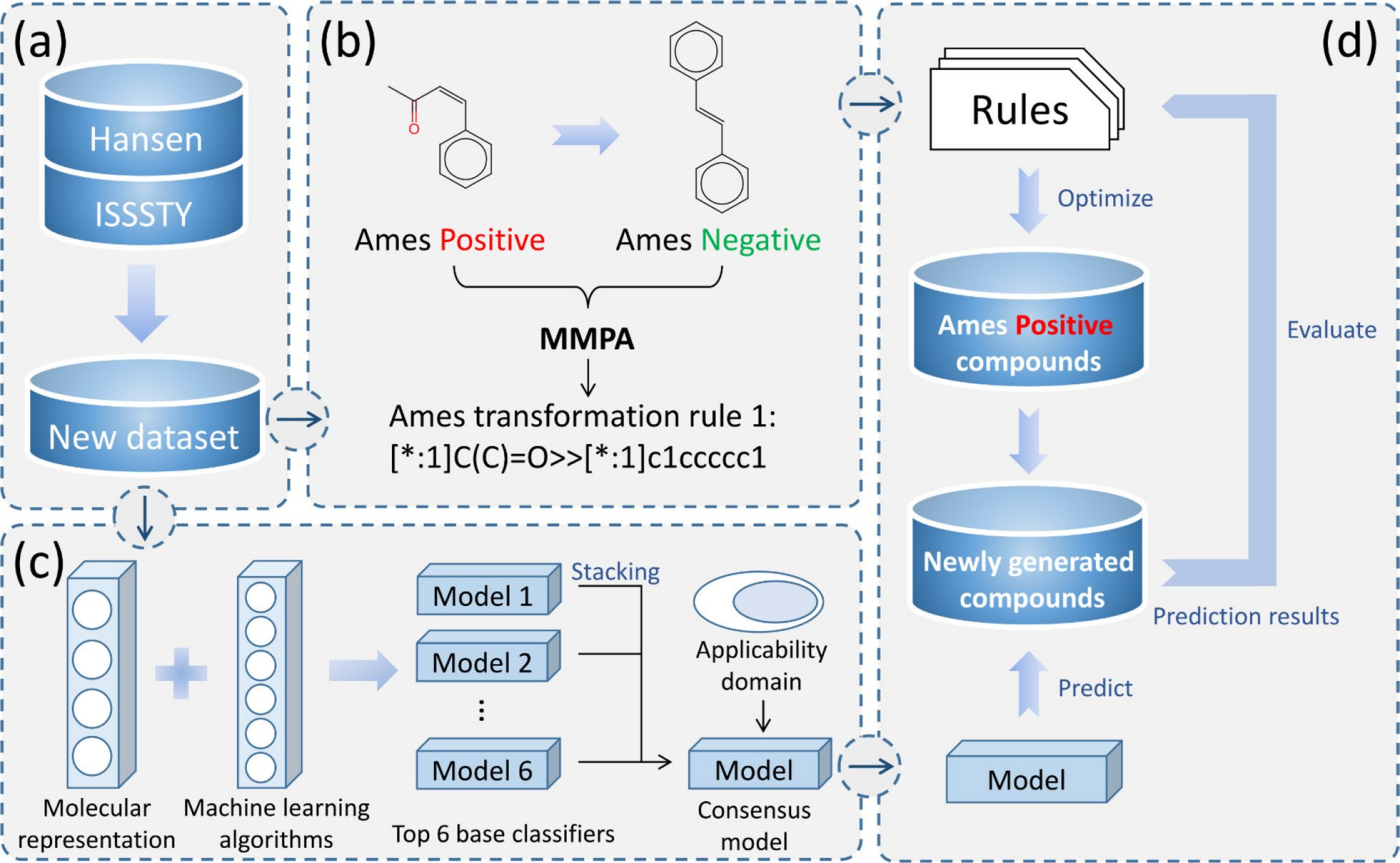
The workflow of this study includes four steps: **a** data collection and preparation, **b** matched molecular pairs analysis to derive mutagenicity transformation rules, **c** the construction of machine learning models for mutagenicity prediction, and **d** evaluation of mutagenicity transformation rules via machine learning models

In this study, we derived and evaluated chemical rules for the optimization of chemical mutagenicity via MMPA and machine learning methods, respectively. As shown in Fig. 1, we first integrated a new Ames mutagenicity data set with structural diversity (Fig. 1a). Then, on the basis of the new data set, we derived mutagenicity transformation rules through MMPA (Fig. 1b) and constructed a machine learning model with a well-defined applicability domain for mutagenicity prediction (Fig. 1c). Subsequently, we evaluated these rules by applying them to the optimization of Ames positives and scoring with a machine learning model (Fig. 1d). Finally, three important factors that might influence the validity of mutagenicity transformation rules were analyzed.

## Materials and methods

### Data collection and preparation

Chemicals were evaluated for their genotoxic potential based on the results in the *Salmonella* bacterial mutagenicity assay, either in the presence or absence of the S9 mix. A compound was judged as Ames positive if it significantly induced revertant colony growth in at least one strain. Only if it did not induce revertant colony growth in any reported strains, it could be regarded as Ames negative. The initial records of Ames mutagenicity data were collected from literature [23] and a publicly accessible database from OECD QSAR Toolbox, i.e., the bacterial mutagenicity ISSSTY database [24]. To evaluate the generalizability of the machine learning models and the detected chemical rules, we included the approved drugs from DrugBank [25] as Ames negative samples, combining with the Ames strong positive data from the Division of Genetics and Mutagenesis, National Institute of Health Sciences (DGM/NIHS) [26] as an external validation set. Notably, these approval drugs involved in this study displayed no mutagenicity or there was no evidence to prove their mutagenicity.

The initial dataset was then curated as follows. All compounds were first converted into canonical SMILES format. Then, mixtures and inorganic compounds were removed, and salts were converted into corresponding acids or bases by Pipeline Pilot Software 2017 R2 (BIOVIA, USA). The records of duplicate compounds would be re-analyzed and assigned new labels. In addition, the same records as the DGM/NIHS data set were removed. Subsequently, we assigned the Ames data randomly into a training set and a test set with a ratio of 9:1. In cross-validation, the training set would be subdivided into a new training set and a validation set according to the number of folds.

### Python-based matched molecular pairs analysis

The detection of MMPs and the generation of transformation rules were implemented based on the Hussain and Rea algorithm [27], which had been codified as an open-source python package termed mmpdb [28]. As shown in Fig. 1b, only the transformation rules that were extracted from Ames positives to Ames negatives were regarded as mutagenicity transformation rules. The detected rules were encoded into SMIRKS format. To better define MMPs, the changing fragments of a molecule were limited between 2 and 15 heavy atoms. The portion of heavy atoms in changing fragments was no more than half of the molecule. In addition, multiple cuts, including single-cut, double-cut and triple-cut, were performed to obtain all possible fragments of a molecule. The chirality was preserved when cutting a bond. In general, different types of fragments would be generated with different cutting methods, which determined the category of the transformation rules. The fragments generated with single-cut, double-cut and triple-cuts were defined as side chains, linkers, and scaffolds, respectively. Finally, the local environment of the attachment points was calculated and recorded in a SHA256 hash of the circular fingerprints with a radius from 0 to 5. Notably, we can obtain multiple mutagenicity transformation rules from one MMP, and one mutagenicity transformation rule can be extracted from different MMPs.

### Construction of machine learning models

#### Molecular representation

A total of four types of molecular representation methods were used to represent the structural features of the molecules in this study, including three molecular fingerprints and one molecular graph. The MACCS fingerprints (MACCS, 166 bits), RDK fingerprints (RDK, 2048 bits), and extended connectivity fingerprints with a radius of 2 continuous bonds and a length of 1024 bits (ECFP, 1024 bits) were generated with the RDKit package (version 2021.03.4) [29]. The molecular graph integrated nine types of atomic features for each atom as node features, and four types of bond features for each bond as edge features, to construct an initial vector. The RDKit package (version 2021.03.4) was performed to calculate both atomic features and bond features.

#### Model construction

Six popular machine learning algorithms, including SVM [30], RF [13], extreme gradient boosting (XGBoost) [31], light gradient boosting machine (LightGBM) [32], gradient boosting (GB) [33], and a graph neural network algorithm (GNN) named Attentive FP [16], were employed to develop the base classifiers for chemical mutagenicity prediction. Except for the Attentive FP algorithm, the hyper-parameters involved in the mentioned algorithms were optimized using the fivefold cross-validation and grid search. The Attentive FP algorithm adopted the Bayesian optimization for hyper-parameters search and the Adam optimizer for gradient descent optimization. To avoid overfitting, we applied an early stop strategy. The training process would be terminated early if the area under curve (AUC) values had not improved in 8 epochs on the training set and 10 epochs on the validation set. To verify the robustness of the GNN model, we utilized the tenfold cross-validation.

To make full use of these base classifiers and improve the predictive capability of the final model, we used a model stacking strategy to construct a consensus model. The core idea of the strategy was to integrate the predicted probabilities from different base classifiers into a feature matrix and retrain them to generate a new model. Here, the logistic regression algorithm (LR) [34] was performed to develop a consensus model based on the six best base classifiers.

The SVM, RF, GB, and LR algorithms were implemented using the scikit-learn package (version 1.0). The XGBoost and LightGBM algorithms were implemented using the xgboost package (version 1.5.2) and the lightgbm package (version 3.3.2), respectively. The Attentive FP algorithm was implemented using Xiong’s code [16].

#### Model evaluation

The validation set and test set were used to evaluate the performance of each model. Five statistical indexes, namely AUC, accuracy (ACC), sensitivity (SE), specificity (SP), and F1-score (F1), were calculated. The equations of these indexes were given in Additional file 1: Table S1. The AUC and F1 values can characterize the overall performance of the model. SE, equivalent to recall, measures the predictive ability of the model for positive samples. On the contrary, SP represents the model’s prediction ability of negative samples. Generally, SE is more important than SP in toxicity prediction because detecting more compounds with potential toxic can effectively reduce the cost in early drug discovery.

#### Definition of applicability domain

Limited by the chemical space of compounds in the training set, each machine learning model was biased towards predicting a specific type of compounds, i.e., applicability domain. That is to say, the prediction results were more reliable if the predicted compounds were within the applicability domain of a specific machine learning model. In this study, a similarity-based method [35] was used to determine the applicability domain of the consensus model. We first calculated the Tanimoto similarity indexes with ECFP between a given compound and each compound in the training set, where the top *K* Tanimoto similarity indexes were regarded as the similarity of the given compound to the training set. Then, we searched for the best similarity threshold (D_T_), where compounds within the threshold should have better and more reliable prediction results. The definition of similarity threshold was shown in Eq. 1. The equation had two hyper-parameters, *K* and *z*. The grid search was performed to determine the optimal *K* and optimal *z*.

1 $$D_{T} = \overline{\gamma } + z\sigma$$

In this equation, ${D}_{T}$ represents the similarity threshold of the model, i.e., the applicability domain. $\overline{\gamma }$ represents the average Tanimoto similarity index of the compounds in the training set and $\sigma$ is the standard derivation of the Tanimoto similarity index of all the compounds in the training set. z is a hyper-parameter representing the significance level. For a given compound, if the Tanimoto similarity indexes of its *K* most similar molecules all exceed the defined similarity threshold D_T_, it is regarded as in domain (ID), otherwise out of the domain (OD).

### Evaluation of mutagenicity transformation rules with machine learning models

We first assumed that the mutagenicity transformation rules detected with MMPA could be used in the optimization of other Ames positives. Then, we integrated all Ames positives into a new data set and transformed them with MMP rules. The newly generated compounds would be predicted with the well-trained consensus model. Each mutagenicity transformation rule could be used for the optimization of many Ames positives and each Ames positive compound could be optimized with different mutagenicity transformation rules. To evaluate the applicability and reliability of each obtained mutagenicity transformation rule, we defined an evaluation metric, namely S_Validity_ (Eq. 2). It evaluated the validity of a given mutagenicity transformation rule by calculating the proportion of the newly generated compounds that were predicted to be Ames negative. Notably, only those newly generated compounds within the applicability domain of the consensus model were included in the statistics.

2 $$S_{validity} = \frac{{N_{neg} }}{{N_{total} }}$$

In this equation, ${N}_{neg}$ represents the number of newly generated compounds that are predicted as Ames negative, and ${N}_{total}$ is the number of newly generated compounds.

## Results

### Data set analysis

In this study, we collected the Ames records from Hansen’s benchmark (6512 compounds) [23] and the ISSSTY database (6052 compounds) [24]. After data preparation, a total of 8576 compounds with structural diversity were obtained, including 4643 Ames positives and 3933 Ames negatives. The comprehensive data set was then split into a training set including 7720 compounds and a test set containing 856 compounds. Overall, the numbers of negatives and positives in this data set were balanced with a ratio of 0.847 (Neg./Pos.). In addition, 805 approved drugs from DrugBank [25] that were not involved in the training set, and 664 Ames strong positive samples from DGM/NIHS [26] were built as an external validation set. The numbers and sources of compounds in different data sets were shown in Table 1.

**Table 1:** The number and sources of compounds in different data sets

Data set	Mutagenic	Non-mutagenic	Source
Training set	4174	3546	Hansen’s benchmark & ISSSTY database
Test set	469	387	Hansen’s benchmark & ISSSTY database
External validation set	664	805	DGM/NIHS & DrugBank

**Fig. 2:**
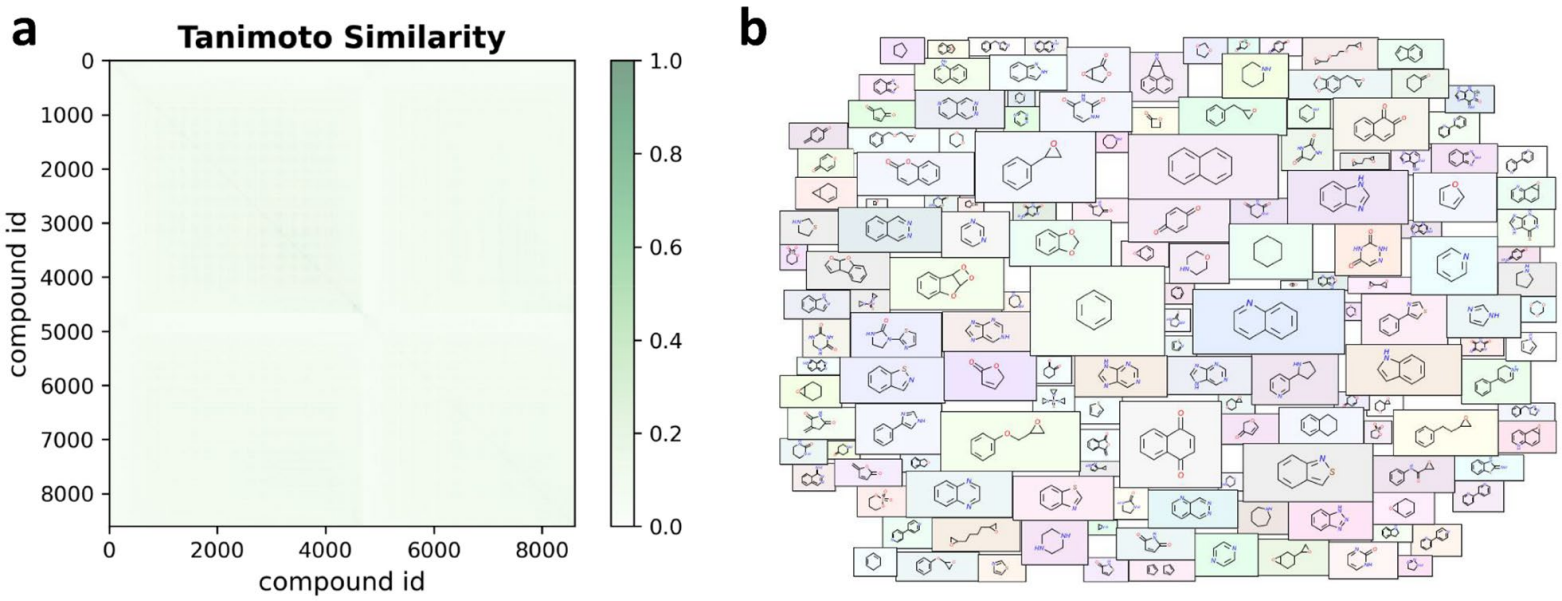
The heat map **a** and the molecular cloud **b** of the Ames data set

To further explore the chemical space of the Ames data set, the Tanimoto similarity indexes and Murcko scaffolds analysis [36] were performed. The Tanimoto similarity indexes were calculated with ECFP and the Murcko scaffolds of the total data set were extracted by removing side chain substituents but retaining the linkers and ring systems with RDKit package [29]. The overall color of the Tanimoto similarity heat map was light green with an average similarity of 0.087 (Fig. 2a), indicating the structural diversity of the data set. Additionally, we detected 1822 different Murcko scaffolds from the data set, suggesting that each Murcko scaffold shared an average of 4.7 molecules. Moreover, more than 80% of the scaffolds were contained in no more than three molecules, indicating a high level of chemical diversity. The molecular cloud [37] was used to visualize the frequency of the detected Murcko scaffold (Fig. 2b). Clearly, molecules with polycyclic scaffolds were the focus of chemical mutagenicity studies. In a word, the above analysis of the Tanimoto similarity indexes and Murcko scaffolds demonstrated the structural diversity of the Ames data set.

### Derivation of mutagenicity transformation rules via MMPA

The MMPA was performed based on the curated Ames data set from Hansen’s benchmark data set and the ISSSTY database. Then, a total of 7485 MMPs and 6107 mutagenicity transformation rules were identified. The total information of all transformation rules was given in Additional file 2: Table S2 (some examples were illustrated in Table 6). The frequency and categories of these transformation rules were summarized in Table 2. Clearly, the single-cut rules had the largest proportion (80.15%) of all the rules while only 37 triple-cut rules were extracted from the data set, which could be attributed to the more restrictive identification conditions of triple-cut rules. It could be observed that structural modification of side chains was the primary scheme for mutagenicity optimization. On the other hand, a large proportion (85.08%) of mutagenicity transformation rules were only detected once from the data set, indicating that there might be some redundant or invalid rules. Furthermore, limited to the amount of Ames data, few double-cut rules and triple-cut rules occurred more than 4 times. In contrast to this, there were some high-frequency single-cut rules, such as “[*:1][N +](= O)[O-] >  > [*:1][H]” and “[*:1]CC1CO1 >  > [*:1][H]”, which had been detected for 172 times and 23 times, respectively. Overall, we successfully extracted mutagenicity transformation rules from the Ames data set with MMPA, and the analysis revealed the important role of the structural modification of side chains in mutagenicity optimization.

**Table 2:** The summary of mutagenicity transformation rules

Frequency	Multiple cuts			Total
	Single-cut	Double-cut	Triple-cut	
1	4114	1053	29	5196 (85.08%)
2–4	704	120	8	832 (13.62%)
5–9	54	2	0	56 (0.92%)
10^+^	23	0	0	23 (0.38%)
Total	4895 (80.15%)	1175 (19.25%)	37 (0.6%)	6107

### Performance of machine learning models on mutagenicity prediction

#### Performance of base classifiers and consensus model

Based on the carefully curated Ames data, we constructed a total of 16 base classifiers for chemical mutagenicity prediction, including 15 conventional machine learning models and a deep learning model. The model performance on the training set was evaluated with cross-validation (Additional file 1: Table S3). For each machine learning method, the best model was selected according to the AUC and SE values of cross-validation. Finally, the RF_RDK, SVM_ECFP, LGB_RDK, XGB_MACCS, GBT_MACCS, and GNN models performed better than the other models and were preserved as the best base classifiers.

**Fig. 3:**
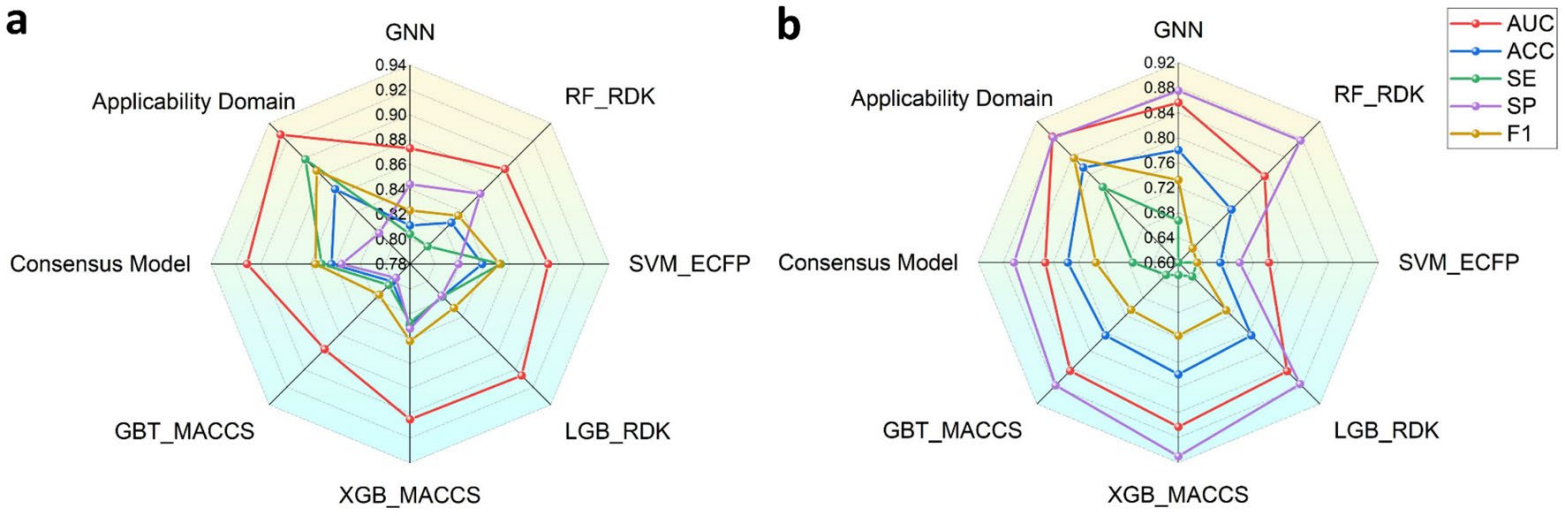
The model performance of six base classifiers (GNN model, RF_RDK model, SVM_ECFP model, LGB_RDK model, XGB_MACCS model and GBT_MACCS model) and consensus model in the test set **a** and external validation set **b**. The ‘Applicability Domain’ referred to the performance of consensus model considering only the compounds within the applicability domain

According to previous quantitative structure-activity relationship (QSAR) studies, the consensus model combining multiple base classifiers tended to have better model robustness and predictive capability [38–40]. Therefore, we applied a model stacking strategy by integrating the prediction probabilities of six base classifiers and fed them into a logistic regression algorithm to generate a consensus model. The well-trained consensus model showed favorable performance in the test set (Additional file 1: Table S4). For a more intuitive comparison, we visualized the performance of the base classifiers and consensus model in the test set and external validation set (Fig. 3). According to the AUC values, the performance of the consensus model was improved by 0.4–4% in test set. In addition, compared with base classifiers, the consensus model also exhibited satisfactory accuracy and sensitivity in the external validation set (Additional file 1: Table S5). Even so, nearly 22% of the compounds in external validation set were still incorrectly predicted by the consensus model, which might be due to the fact that some of these compounds were out of the applicability domain of the model.

#### Determination of applicability domain with test set

A defined applicability domain is one of the five OECD principles for the validation of QSAR models [41]. Molecules within the applicability domain tended to obtain more reliable prediction results. In this study, the test set was used to define the applicability domain. We calculated the average Tanimoto similarity index of the compounds in the training set ($\overline{\gamma }$) of 0.0880 and the standard derivation of the Tanimoto similarity index ($\sigma$) of 0.0625. Then, to minimize the loss of valuable chemical space, we considered both the number of compounds in the test set and the predictive performance of the consensus model to explore optimal k and Z. The final similarity threshold (D_T_) was 0.338 with an optimal k and Z of 5 and 4, respectively. The defined applicability domain contained nearly 80% (679 out of 856) compounds of the test set and obtained a favorable predictive capability of these ID compounds (AUC = 0.927, ACC = 0.865, SE = 0.899, SP = 0.815, F1 = 0.886). As shown in Fig. 3, the model performed better in predicting ID compounds, indicating that the defined applicability domain successfully summarized the model’s preference.

#### Performance on the external validation set

To evaluate the generalizability of the consensus model, we integrated an external validation set with 664 Ames strong positive compounds from DGM/NIHS and 805 approved drugs as Ames negative samples. For the total external validation set, there were 376 positives and 239 negatives within the applicability domain of the consensus model. Overall, our consensus model had an accuracy of 0.815 for the ID compounds. Specifically, 290 (nearly 77.1%) Ames strong positive compounds and 211 (nearly 88.3%) Ames negative samples were correctly predicted.

The Ames strong positive compounds from DGM/NIHS were an external validation set used in the Ames/QSAR International Challenge Project [10]. The initial number of Ames strong positive samples should be 672, but 8 of them had ambiguous incomplete SMILES and were therefore not included in our external validation set. A total of 12 QSAR vendors with 17 QSAR tools, including 11 statistical-based models and 6 rule-based models, participated in the Ames QSAR International Challenge Project. When predicting these Ames strong positive compounds, these statistical-based models and rule-based models obtained an average SE of 0.690 and 0.749, respectively. By contrast, our consensus model had a SE of 0.771, which was better than 91% (10 out of 11) statistical-based models and 66% (4 out of 6) rule-based models (Additional file 2: Table S6). The results demonstrated the strong positive predictive power of our consensus model. In addition, according to the prediction results of Ames negative samples, our consensus model also showed favorable performance.

### Analysis of mutagenicity transformation rules

#### Mutagenicity optimization with mutagenicity transformation rules

The obtained mutagenicity transformation rules could be used in structural transformation of the compounds sharing the same substructures. If a compound had multiple identical substructures, only one of these substructures would be transformed once. To investigate whether these transformation rules could be used for mutagenicity optimization, we first extracted the Ames positives from the Hansen/ISSSTY data set and DGM/NIHS data set and transformed them with MMP rules. Subsequently, the newly generated compounds would be predicted with the well-trained consensus model. Table 3 illustrated the changes in the number of compounds when transformed with mutagenicity transformation rules. For example, among the 664 Ames positives from DGM/NIHS data set, 540 ones could be transformed using the transformation rules and a total of 24311 compounds were generated. After feeding these newly generated compounds into the consensus model, we found that there were 12716 ID compounds, where 7527 ones were predicted as Ames positive and 5189 ones were classified as Ames negative. It was clear that these mutagenicity transformation rules could be used for most Ames positives, which showed the generalization of these rules.

**Table 3:** Changes in the number of compounds when transformed with mutagenicity transformation rules

Dataset	N_pos_	N_trans_	N_gen_	N_ID_ (N_pos._ vs N_neg._)
DGM/NIHS	664	540	24311	12716 (7527 vs 5189)
Hansen/ISSSTY	4670	3213	118677	93663 (63269 vs 30394)

N_pos._: the number of positive compounds. N_trans_: the number of positive compounds that can be transformed with mutagenicity transformation rules. N_gen_: the number of newly generated compounds. N_ID_: the number of newly generated compounds within the applicability domain of the consensus model. N_pos._: the number of compounds that are predicted as Ames positive. N_neg._: the number of compounds that are predicted as Ames negative

**Fig. 4:**
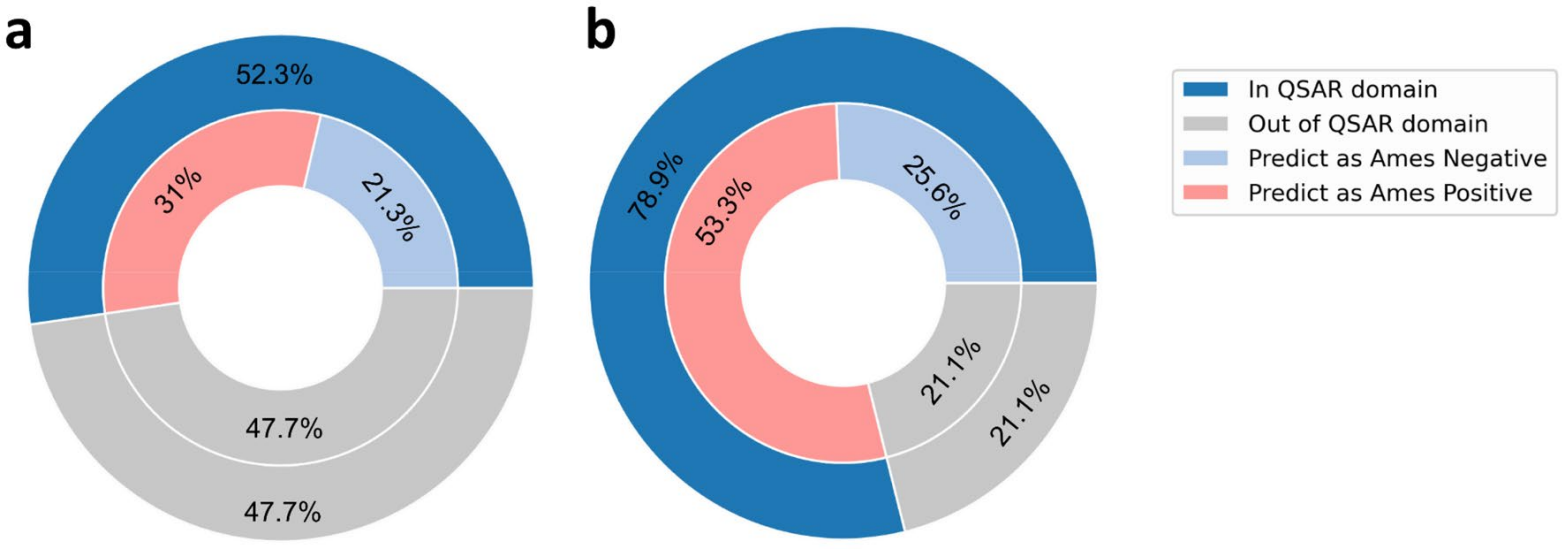
The distribution of the prediction results of newly generated compounds from the DGM/NIHS data set **a** and Hansen/ISSSTY data set **b**

As shown in Fig. 4, Ames negatives occupied a considerable proportion of all the newly generated compounds, indicating that the mutagenicity transformation rules were of great practicability and could be used in mutagenicity optimization. On the other hand, it should be noted that a large amount of newly generated compounds was still predicted as Ames positive, which revealed that there were some invalid transformations. Therefore, an evaluation metric, namely S_Validity_, was defined to evaluate the transformation validity of each mutagenicity transformation rule.

#### Evaluation of mutagenicity transformation rules

In this study, we calculated the S_Validity_ of each mutagenicity transformation rule in two data sets. A total of 1629 and 5612 mutagenicity transformation rules were used in DGM/NIHS data set and Hansen/ISSSTY data set, respectively. The S_validity_ of these rules was summarized in Additional file 2: Table S7. For simplicity, we defined those rules with a S_validity_ higher than 0.5 as high-quality mutagenicity transformation rules. According to the statistics, high-quality transformation rules accounted for 45.3% and 68.3% of all those rules used in mutagenicity optimization of DGM/NIHS data set and Hansen/ISSSTY data set, respectively. Furthermore, given the accidental errors caused by those less frequently used rules, we filtered out those transformation rules that were used less than 10 times in the optimization of Ames positives of two data sets. The proportions of high-quality mutagenicity transformation rules dropped to 28.3% and 36.8%, respectively. In addition, when using these rules in the optimization of Ames positives in the DGM/NIHS data set, the invalid mutagenicity transformation rules (S_validity_ = 0) only accounted for 4.8%, suggesting that most transformation rules could effectively reverse chemical mutagenicity. Overall, the results indicated that the mutagenicity transformation rules were of great practical value in mutagenicity optimization. Nevertheless, it should be noted that these rules might only be applied to specific chemical environments and the abuse of these rules could easily lead to invalid transformations.

#### Factors influencing the performance of mutagenicity transformation rules

In this part, we analyzed three factors that might influence the performance of mutagenicity transformation rules, including local chemical environment, rule frequency and rule category.

The local chemical environment of the attachment points of each transformation rule was encoded into the circular fingerprints with a radius from 0 to 5. When transformed using these rules, one could set the minimum radius to ensure the same local environment at the attachment point. It was easy to appreciate that identical substitution at different molecules might result in different property changes [17, 22]. For example, a transformation rule derived from aromatic compounds might not be applied to aliphatic ones. We then counted and recorded the transformation validity of these rules by calculating the percentage of successfully transformed molecules at different radii (Table 4). Clearly, the transformation validity increased with the local environment radius and the transformation validity reached 88.7% and 94.9% at the local environment radius of 5. The result indicated that the local environment had a great impact on the validity of transformation rules. The larger the local environment radius considered when using these rules, the higher the success rate of mutagenicity optimization.

**Table 4:** Statistics of newly generated compounds from two data sets at different environment radii

Radius	Newly generated compounds from DGM/NIHS data set				Newly generated compounds from Hansen/ISSSTY data set			
	N_gen_	N_neg_	S_t.v._	N_rules_	N_gen_	N_neg_	S_t.v._	N_rules_
0	7751	2928	37.8%	1159	51779	12425	24.0%	2652
1	3178	1280	40.3%	780	22718	6652	29.3%	1793
2	891	451	50.6%	397	7240	2693	37.2%	1108
3	690	409	59.3%	293	4207	2432	57.8%	1103
4	153	74	48.4%	77	2765	1489	53.9%	460
5	53	47	88.7%	49	4954	4703	94.9%	4334

N_gen_: the number of newly generated compounds. N_neg._: the number of compounds that are predicted as Ames negative. S_t.v._: the transformation validity of mutagenicity transformation rules at the different radius. N_rules:_ the number of mutagenicity transformation rules that were used at the different radii

**Fig. 5:**
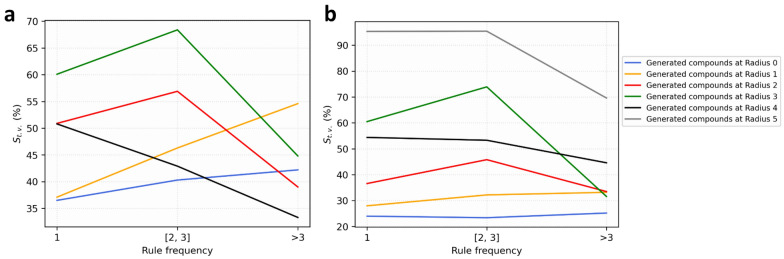
The influence of rule frequency on transformation validity. The S_t.v._ were calculated through DGM/NIHS data set **a** and Hansen/ISSSTY data set **b**, respectively

Rule frequency was an important parameter in some MMP rules-related studies [42, 43]. In this study, we divided these mutagenicity transformation rules into three categories based on rule frequency, then calculated the transformation validity of each category in different local environment radii (Additional file 2: Table S8). As shown in Fig. 5, it was surprising that high-frequency rules (Frequency > 3) did not perform better than the other rules. By contrast, those rules with the frequency of 2 and 3 could transform more efficiently in most scenarios. We further analyzed a high-frequency rule “[*:1][N +](= O)[O-] >  > [*:1][H]” and found that the transformation of the nitro group into a hydrogen atom did not necessarily reverse the mutagenicity, because the nitro group was not necessarily responsible for mutagenicity [44]. Therefore, we speculated that the rule frequency was just an extrinsic property of mutagenicity transformation rules, depending on the chemical space that the experimental researchers focused on, and it was not directly related to the transformation validity.

Different fragmentation protocols would generate different categories of MMP rules, including single-cut, double-cut and triple-cut rules. Here, we recorded the transformation results of different rule categories at different local environment radii (Additional file 2: Table S9). According to Table 5, the single-cut rules performed better than the double-cut rules, indicating that the structural modification of the side chain was more efficient than that of the linker in mutagenicity optimization. In addition, the local chemical environment of triple-cut rules was more complicated than those of single-cut rules and double-cut rules, which narrowed the applicability domain of the triple-cut rules. Thus, only 83 compounds were generated from Hansen/ISSSTY data set with triple-cut rules. Nevertheless, the transformation validity of triple-cut rules was no less than 50% across all the local environment radii, which indicated that the structural modification of molecule scaffolds was a feasible approach in mutagenicity optimization and might even yield better results.

**Table 5:** Statistics of newly generated compounds using different categories of rules from two data sets

Rule Category	Newly generated compounds from DGM/NIHS data set				Newly generated compounds from Hansen/ISSSTY data set			
	N_gen_	N_neg_	S_t.v._	N_rules_	N_gen_	N_neg_	S_t.v._	N_rules_
Single-cut	11715	4995	42.6%	1511	80637	26614	33.0%	4568
Double-cut	1005	194	19.3%	118	12943	3712	28.7%	1017
Triple-cut	–	–	–	–	83	68	81.9%	27

## Discussion

Toxicity has always been a field of great concern for medicinal chemists in lead optimization [45, 46]. In this study, we derived and evaluated the chemical rules for mutagenicity optimization via MMPA and machine learning methods, respectively. With MMPA, we derived those MMP rules from Ames positives to Ames negatives and explored whether these rules could be applied in mutagenicity optimization. Furthermore, to evaluate the applicability and reliability of mutagenicity transformation rules, we constructed a machine learning model with a well-defined applicability domain and favorable performance (Fig. 3). Through the complementarity of MMPA and machine learning models, we summarized a series of valuable mutagenicity transformation rules (Additional file 2: Table S2), which might provide new clues for mutagenicity optimization.

To avoid potential mutagenicity of drug candidates in early drug discovery, experts summarized a series of structural patterns whose presence may induce mutagenicity, i.e. structural alerts (SAs) [47]. For example, the aromatic amino group was considered as a SA for mutagenicity, because the amino group was easily transformed into nitrogen ions to react with DNA [48]. In this study, we detected a series of valuable mutagenicity transformation rules. As shown in Table 6, these rules obtained satisfactory results in mutagenicity optimization. More importantly, the functional groups in these rules were SAs that had been reported before. For example, the aliphatic halogens (Rule ID: 1504) and nitrosamine groups (Rule ID: 1605, 724, 4267, 681) were mutagenicity-related SAs included in ToxAlerts [49]. From this point, our mutagenicity transformation rules provided a promising alternative for the substitution of SAs.

**Table 6:**
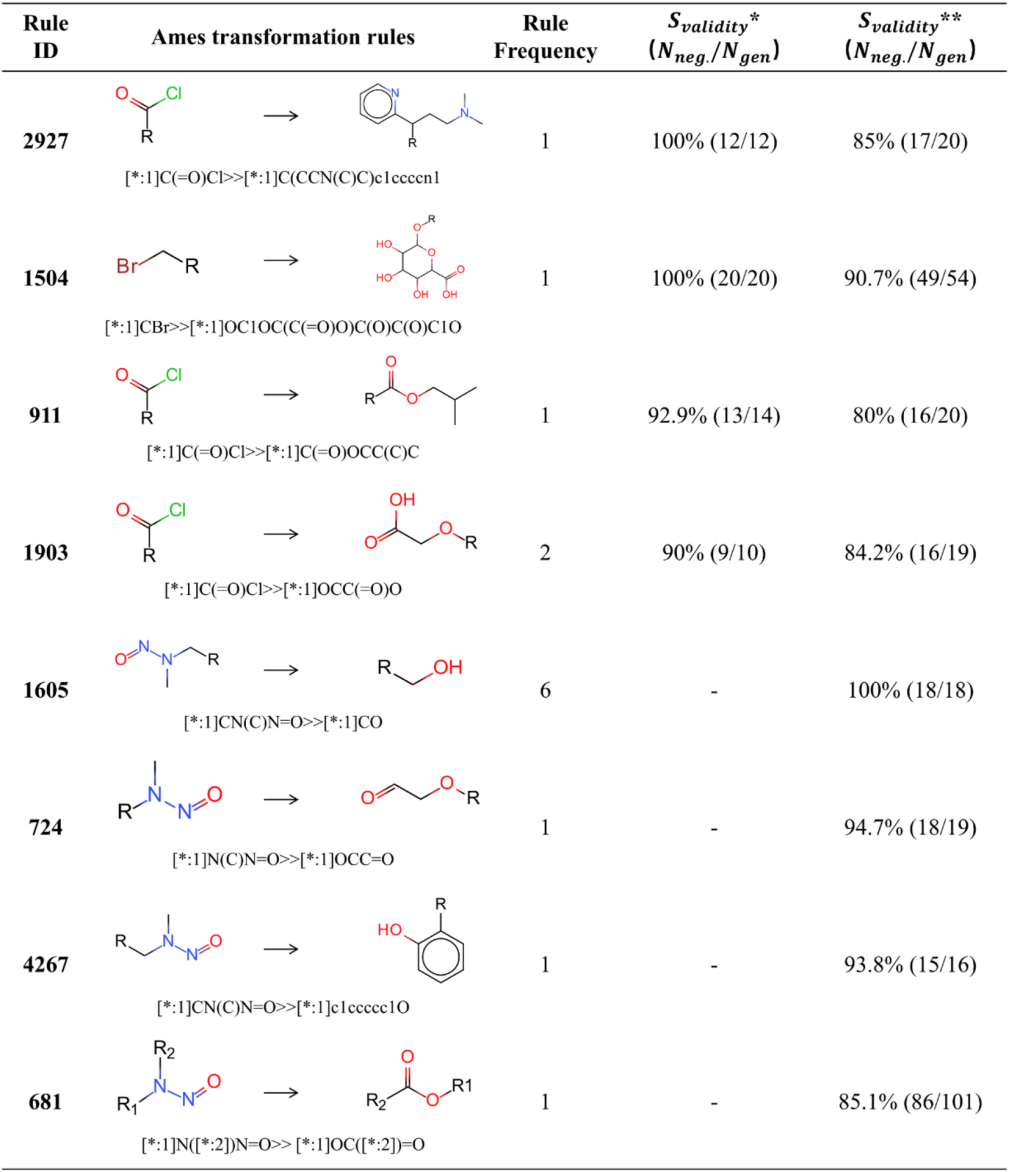
Mutagenicity transformation rules for the substitution of structural alerts

*S*_validity_* was calculated through the transformation of Ames positives from the DGM/NIHS data set. *S*_validity_** was calculated through the transformation of Ames positives from the Hansen/ISSSTY data set.

In previous studies, researchers evaluated MMP rules by accessing the significance of the difference in the property changes with different statistical tests [22, 50]. However, this evaluation method was limited by the data type of the property of interest and the frequency of MMP rules. For example, Fu et al. evaluated the chemical transformation rules for logD7.4 values with Wilcoxon signed-rank test but it could be only performed in those rules that were presented in more than 10 MMPs [50]. Clearly, this method filtered out many valuable but low-frequency MMP rules. In this study, we evaluated the mutagenicity transformation rules by applying them to optimize Ames positives and scoring with a well-trained machine learning model. In theory, each transformation rule could obtain a S_validity_ value to represent the reliability of the rule. In this way, we demonstrated the generalizability of these valuable mutagenicity transformation rules.

**Fig. 6:**
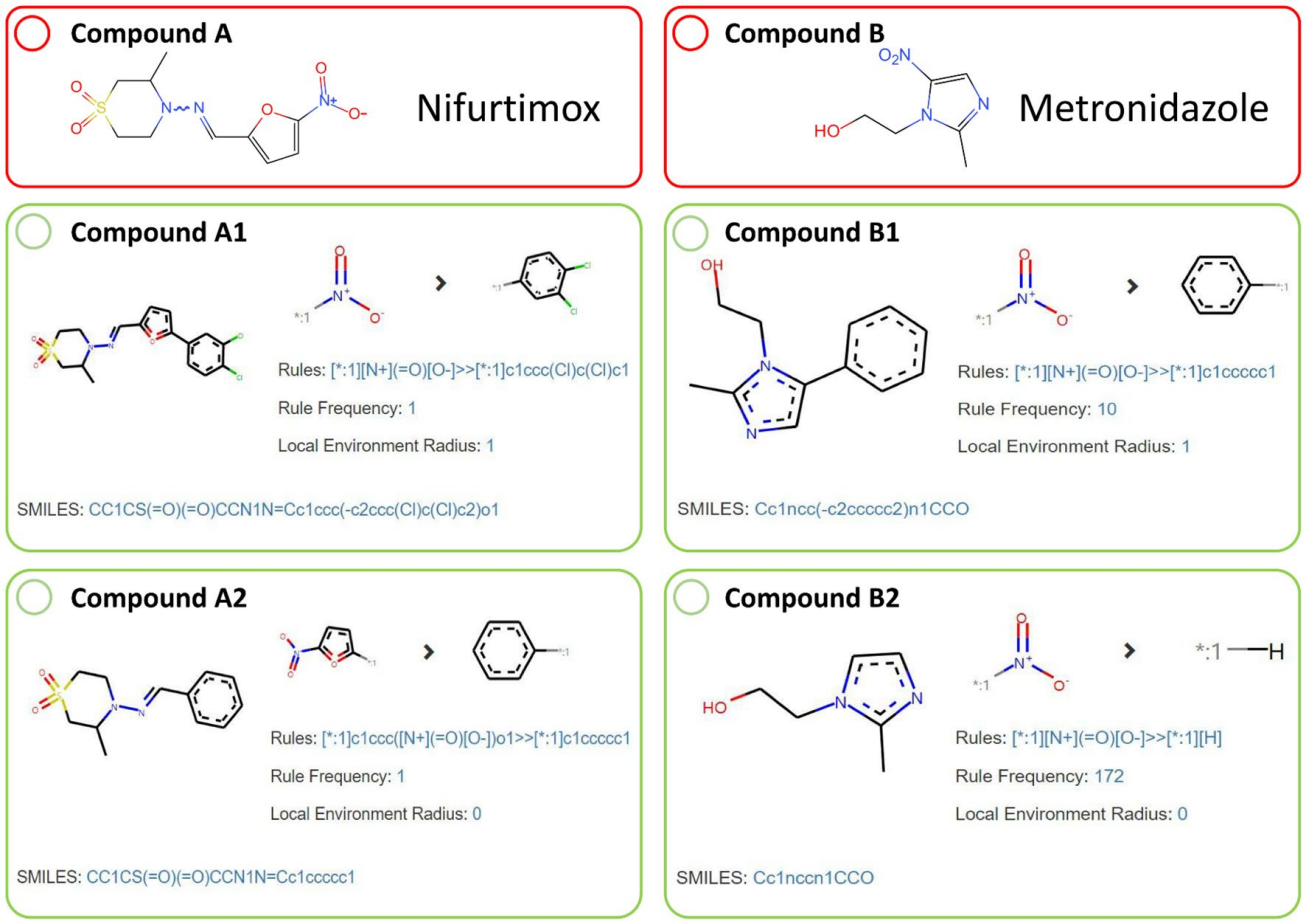
The structures of nifurtimox (compound **A**) and metronidazole (compound **B**), and the newly generated compounds (compounds A1, A2, and B1, B2 from the optimization of nifurtimox and metronidazole, respectively). The compounds in red boxes were predicted as Ames positive, and the ones in green boxes were predicted as Ames negative

To make better use of these mutagenicity transformation rules, we integrated them into ADMETopt2 (http://lmmd.ecust.edu.cn/admetsar2/admetopt2/), a free web server for optimization of chemical pharmacokinetics properties and toxicity. Two drugs, i.e., nifurtimox and metronidazole, which had been reported to have potential mutagenic effects [51–53], were used as case studies to prove the practicability of these rules. We first predicted these two drugs with our machine learning model and the prediction results also illustrated that they were Ames positives. Then, we used mutagenicity transformation rules to explore the structural modification schemes to reverse the mutagenicity of these two drugs. As described in Fig. 6, we obtained some new chemical entities which were predicted to be Ames negative. In this way, medicinal chemists could get more inspiration for mutagenicity optimization. Moreover, to get new chemical entities with favorable ADMET properties, several ADMET models in our admetSAR 2.0 system [54] (http://lmmd.ecust.edu.cn/admetsar2/) could be used to narrow the chemical space of the newly generated compounds. Therefore, we provided a user-friendly platform for the use of these valuable mutagenicity transformation rules.

However, there were still several limitations of these transformation rules. First, these transformation rules could fix potential mutagenicity issues but might result in unexpected changes in other properties of interest. Second, some of the transformation rules would cause large structural changes in a given compound. Finally, there were some incorrect or invalid transformation rules in thee current set of rules. We are actively developing computational methods to solve these potential limitations, for example, using QSAR models to remove newly generated compounds with poor properties and using 3D similarity changes of molecules to select the most appropriate rules. Meanwhile, we are deeply aware that computational algorithms and expert systems are indispensable to obtaining a larger number of transformation rules with better quality. We will continue to update the mutagenicity data set to obtain more effective transformation rules and learn from expert systems to optimize the existing rules.

## Conclusions

The optimization of chemical mutagenicity is of great significance in lead optimization. In this study, we derived mutagenicity transformation rules from a curated Ames mutagenicity data set with MMPA method and evaluated them with a well-trained consensus model. We demonstrated the generalizability and validity of these mutagenicity transformation rules and analyzed three important factors that might influence the validity of the mutagenicity transformation rules. To make better use of these rules, we integrated them into our free web server named ADMETopt2 (http://lmmd.ecust.edu.cn/admetsar2/admetopt2/). Overall, this study provides a new avenue to reverse chemical mutagenicity of compounds, and the strategy can be extended to the optimization of other toxicity endpoints.

## Supplementary Information

**Additional file 1: Table S1.** Equations of three statistical indexes. **Table S3.** Cross-validation results of 16 base classifiers. **Table S4.** The performance of six base classifiers and consensus model in test set. **Table S5.** The performance of six base classifiers and consensus model in external validation set.

**Additional file 2: Table S2.** The information of obtained mutagenicity transformation rules. **Table S6.** The sensitivity of 12 QSAR tools in Ames QSAR International Challenge Project when predicting Ames strong positive compounds. **Table S7.** The S_validity_ of mutagenicity transformation rules that are used in the DGM/NIHS data set and Hansen/ISSSTY data set. **Table S8.** The statistics of mutagenicity transformation rules with different rule frequencies that are used in the DGM/NIHS data set and Hansen/ISSSTY data set. **Table S9.** The statistics of different categories of mutagenicity transformation rules that are used in the DGM/NIHS data set and Hansen/ISSSTY data set.

## Data Availability

All data involved in this study are available in addition file. In addition, the relevant data and code for mutagenicity prediction are available at http://github.com/Louchaofeng/Ames-mutagenicity-optimization. The commercial software platform Pipeline Pilot was purchased by the East China University of Science and Technology and licensed from BIOVIA (https://www.3ds.com/products-services/biovia/products/data-science/pipeline-pilot/). The free software, including RDKit (http://www.rdkit.org), SciPy (http://www.scipy.org), Scikit-learn (https://scikit-learn.org/), and MMPDB (https://github.com/rdkit/mmpdb) are freely available at their websites. The Attentive FP algorithm is available at OpenDrugAI (https://github.com/OpenDrugAI/AttentiveFP).

## Abbreviations

RF: Random forest
SVM: Support vector machine
MMPA: Matched molecular pairs analysis
MMP: Matched molecular pair
DGM/NIHS: Division of genetics and mutagenesis, national institute of health sciences
MACCS: MACCS fingerprints
RDK: RDK fingerprints
ECFP: Extended connectivity fingerprints
XGBoost: Extreme gradient boosting
LightGBM: Light gradient boosting machine
GB: Gradient boosting
GNN: Graph neural network
AUC: Area under curve
LR: Logistic regression
ACC: Accuracy
SE: Sensitivity
SP: Specificity
F1: F1-score
ID: In domain
OD: Out of domain
QSAR: Quantitative structure–activity relationship
SA: Structural alert
