# Supplementary material for: Chemical rules for optimization of chemical mutagenicity via matched molecular pairs analysis and machine learning methods
Source: J Cheminform. 2023 Mar 20;15:35. doi: 10.1186/s13321-023-00707-x (PMC10029263; doi:10.1186/s13321-023-00707-x)
Supplement: Supplementary file 1 — Additional file 1: Table S1. Equations of three statistical indexes. Table S3. Cross-validation results of 16 base classifiers. Table S4. The performance of six base classifiers and consensus model in test set. Table S5. The performance of six base classifiers and consensus model in external validation set. [file 13321_2023_707_MOESM1_ESM.docx]

**Chemical Rules for Optimization of Ames Mutagenicity via Matched Molecular Pairs Analysis and Machine Learning Methods**

Chaofeng Lou, Hongbin Yang, Hua Deng, Mengting Huang, Weihua Li, Guixia Liu, Philip W. Lee, Yun Tang*

*Shanghai Frontiers Science Center of Optogenetic Techniques for Cell Metabolism, School of Pharmacy, East China University of Science and Technology, Shanghai 200237, China*

*Corresponding author. E-mail: [ytang234@ecust.edu.cn](mailto:ytang234@ecust.edu.cn).

**Table Legends**

**Table S1.** Equations of three statistical indexes

**Table S3.** Cross-validation results of 16 base classifiers

**Table S4.** The performance of six base classifiers and consensus model in test set

**Table S5.** The performance of six base classifiers and consensus model in external validation set

**Table S1.** Equations of three statistical indexes

| **Index** | **Name** | **Equation** |
| --- | --- | --- |
| 1 | Accuracy | $\frac{TP+TN}{TP+TN+FP+FN}$ |
| 2 | Sensitivity | $\frac{TP}{TP+FN}$ |
| 3 | Specificity | $\frac{TN}{TN+FP}$ |
| 4 | F1-Score | $\frac{2TP}{TP+FN+TP+FP}$ |

In this study, we evaluated machine learning models through confusion matrix. In this equations, TP represents the number of positive samples that are correctly predicted, TN is the number of negative samples that are correctly predicted, FP represents the number of positive samples that are wrongly predicted, FN is the number of negative samples that are wrongly predicted.

**Table S3.** Cross-validation results of 16 base classifiers

| **Model** | **AUC** | **ACC** | **SE** | **SP** | **F1** |
| --- | --- | --- | --- | --- | --- |
| GNN | 0.877±0.012 | 0.812±0.013 | 0.828±0.023 | 0.827±0.020 | 0.827±0.012 |
| RF_MACCS | 0.89±0.007 | 0.817±0.004 | 0.811±0.008 | 0.825±0.012 | 0.828±0.003 |
| RF_RDK | 0.898±0.005 | 0.825±0.003 | 0.828±0.008 | 0.823±0.007 | 0.837±0.004 |
| RF_ECFP | 0.881±0.007 | 0.804±0.005 | 0.787±0.016 | 0.824±0.012 | 0.813±0.007 |
| SVM_MACCS | 0.9±0.008 | 0.839±0.008 | 0.845±0.008 | 0.831±0.014 | 0.851±0.007 |
| SVM_RDK | 0.894±0.008 | 0.832±0.008 | 0.842±0.012 | 0.821±0.021 | 0.845±0.007 |
| SVM_ECFP | 0.902±0.009 | 0.832±0.012 | 0.862±0.008 | 0.797±0.026 | 0.848±0.01 |
| LGB_MACCS | 0.908±0.007 | 0.836±0.01 | 0.858±0.008 | 0.81±0.014 | 0.851±0.009 |
| LGB_RDK | 0.909±0.005 | 0.84±0.006 | 0.856±0.008 | 0.821±0.015 | 0.853±0.005 |
| LGB_ECFP | 0.899±.009 | 0.828±0.01 | 0.842±0.013 | 0.811±0.022 | 0.841±0.009 |
| XGB_MACCS | 0.906±0.007 | 0.834±0.009 | 0.859±0.003 | 0.804±0.018 | 0.849±0.007 |
| XGB_RDK | 0.906±0.007 | 0.84±0.006 | 0.858±0.01 | 0.819±0.014 | 0.853±0.005 |
| XGB_ECFP | 0.895±0.007 | 0.823±0.012 | 0.831±0.013 | 0.814±0.026 | 0.836±0.01 |
| GBT_MACCS | 0.878±0.007 | 0.811±0.008 | 0.845±0.005 | 0.771±0.021 | 0.829±0.005 |
| GBT_RDK | 0.883±0.005 | 0.82±0.006 | 0.834±0.015 | 0.802±0.011 | 0.834±0.007 |
| GBT_ECFP | 0.874±0.007 | 0.802±0.01 | 0.807±0.018 | 0.797±0.017 | 0.816±0.01 |

**Table S4.** The performance of six base classifiers and consensus model in test set

| **Model** | **AUC** | **ACC** | **SE** | **SP** | **F1** |
| --- | --- | --- | --- | --- | --- |
| GNN | 0.873 | 0.811 | 0.804 | 0.844 | 0.823 |
| RF_RDK | 0.888 | 0.827 | 0.800 | 0.860 | 0.835 |
| SVM_ECFP | 0.891 | 0.838 | 0.853 | 0.819 | 0.852 |
| LGB_RDK | 0.907 | 0.817 | 0.817 | 0.816 | 0.830 |
| XGB_MACCS | 0.905 | 0.829 | 0.827 | 0.832 | 0.842 |
| GBT_MACCS | 0.877 | 0.800 | 0.804 | 0.796 | 0.815 |
| Consensus Model | 0.911 | 0.843 | 0.851 | 0.835 | 0.856 |
| Applicability Domain | 0.927 | 0.865 | 0.899 | 0.815 | 0.886 |

Note: The ‘Applicability Domain’ referred to the performance of consensus model considering only the compounds within the applicability domain.

**Table S5.** The performance of six base classifiers and consensus model in external validation set

| **Model** | **AUC** | **ACC** | **SE** | **SP** | **F1** |
| --- | --- | --- | --- | --- | --- |
| GNN | 0.856 | 0.780 | 0.667 | 0.875 | 0.732 |
| RF_RDK | 0.795 | 0.720 | 0.532 | 0.876 | 0.632 |
| SVM_ECFP | 0.745 | 0.667 | 0.630 | 0.698 | 0.630 |
| LGB_RDK | 0.846 | 0.765 | 0.631 | 0.875 | 0.708 |
| XGB_MACCS | 0.863 | 0.779 | 0.620 | 0.910 | 0.717 |
| GBT_MACCS | 0.845 | 0.765 | 0.628 | 0.878 | 0.707 |
| Consensus Model | 0.813 | 0.777 | 0.673 | 0.863 | 0.732 |
| Applicability Domain | 0.885 | 0.815 | 0.771 | 0.883 | 0.836 |

Note: The ‘Applicability Domain’ referred to the performance of consensus model considering only the compounds within the applicability domain.
